# Outcomes of Intramedullary Nailing Versus Plate Fixation in the Management of Humeral Shaft Fractures: A Systematic Review and Meta-Analysis

**DOI:** 10.7759/cureus.72473

**Published:** 2024-10-27

**Authors:** Adeel Nadeem, Hannah Abbasi

**Affiliations:** 1 Trauma Sciences, Blizzard Institute, Queen Mary University of London, London, GBR; 2 Orthopaedics, Royal National Orthopaedic Hospital, London, GBR; 3 Internal Medicine, Lewisham and Greenwich National Health Service (NHS) Trust, London, GBR

**Keywords:** humeral shaft fractures, humerus shaft, intramedullary nail, open reduction and internal fixation with a plate (orif with plate), orthopaedic surgery, perioperative outcomes, surgical complication

## Abstract

This systematic review and meta-analysis aimed to compare the outcomes of intramedullary nailing (IMN) and open reduction with internal fixation (ORIF) in treating traumatic humeral shaft fractures in adults (18 years and above). A comprehensive literature search was conducted in databases including PubMed, Google Scholar, Embase, and Cochrane Central Register of Controlled Trials (CENTRAL). The primary outcome was time to union, while secondary outcomes included non-union rates, incidences of iatrogenic radial nerve palsy, surgical site infections, and intra-operative comminution. Twenty-six studies, encompassing 1,674 patients (867 IMN, 807 ORIF), were included. IMN demonstrated a shorter time to union compared to ORIF (mean difference -1.06 (95% CI, -1.88 to -0.23)), with significant statistical heterogeneity (I² = 70%), and a mean difference of -2.14 (95% CI, -3.16 to -1.12) in the randomized controlled trial (RCT) subgroup analysis, which had no significant statistical heterogeneity. Both techniques had comparable non-union rates (risk ratio 0.34 (95% CI, 0.94 to 1.93)). IMN was associated with lower incidences of iatrogenic radial nerve palsy (risk ratio 0.48 (95% CI, 0.27 to 0.87)) and surgical site infections (risk ratio 0.44 (95% CI, 0.25 to 0.76)), but had a higher risk of intra-operative comminution (risk ratio 3.04 (95% CI, 1.24 to 7.44)). The studies exhibited significant heterogeneity and varying outcome measures, highlighting the need for cautious interpretation. IMN offers rapid fracture stabilization and minimal additional physiological insult, while ORIF remains preferable for achieving precise anatomical reduction. These findings highlight the importance of considering patient-specific factors and surgical expertise in selecting the appropriate fixation technique.

## Introduction and background

Humeral shaft fractures constitute a significant portion of orthopaedic injuries, accounting for approximately 3% of all fracture presentations, with an annual incidence of 15 per 100,000 individuals [1,2]. These fractures are often associated with substantial impairment in upper limb function and pain, leading to a decline in the quality of life for affected patients during the treatment period. The primary objectives of treatment are to alleviate pain, restore limb function, and improve overall quality of life.

The cornerstone of treatment for humeral shaft fractures is conservative management. This typically involves the use of humeral bracing techniques such as functional bracing, hanging casts, and shoulder spica casts, which collectively achieve union rates as high as 90% [1,3]. Despite this, non-union, malunion, and delayed union rates can be significant, with some studies reporting rates as high as 30% [4-7]. Additionally, conservative management often necessitates prolonged upper limb immobilisation, which can be inappropriate for certain patient populations [3].

Surgical intervention is indicated in specific scenarios, including poly-traumatised patients, neurovascular injury, significant displacement, bilateral injuries, open fractures, and cases where conservative management has failed [1,8]. There is a growing trend towards surgical treatment to facilitate early mobilisation and functional recovery, particularly in younger patients, and with the advancement of surgical techniques and fixation devices, more fracture patterns are considered for fixation by orthopaedic surgeons [1,9].

Among the surgical options, intramedullary nailing (IMN) and open reduction with internal fixation (ORIF) using plates and screws are the two most widely utilised techniques. ORIF, the traditional method, ensures anatomical reduction and has been associated with higher union rates in the literature [10]. However, it involves extensive soft tissue dissection, which increases the risk of infection and radial nerve injury [11].

In contrast, IMN offers a minimally invasive approach that preserves fracture biology through a load-sharing construct, promoting early mobilisation [1,12]. Despite these advantages, IMN is associated with intra-operative risks such as fracture comminution and complications related to the entry points, including pain, stiffness, and impingement due to rotator cuff or elbow joint disruption from antegrade and retrograde insertions, respectively. These complications are not typically associated with ORIF [9].

Despite the benefits of each fixation technique, there remains ongoing debate regarding the optimal surgical method for the stabilisation of humeral shaft fractures. 

Aims and objectives

The overall aim of this systematic review and meta-analysis was to compare the outcomes of IMN and ORIF with plate and screw fixation in the treatment of humeral shaft fractures. Additionally, the secondary aims were to assess the quality of the evidence supporting each fixation modality and to characterise the patient populations managed with either technique in the reported literature.

The main objective was to determine which operative technique is associated with better post-operative outcomes. The secondary objectives were to evaluate the surgical risks associated with each technique to better inform consenting practices and to assess the quality of evidence supporting current practice, highlighting areas with a paucity of evidence for future research.

## Review

Methodology

The review protocol outlined was registered prospectively on the PROSPERO International Prospective Register of Systematic Reviews (CRD42024501686). 

This systematic review was based on the Cochrane Handbook for Systematic Reviews of Interventions [13]. The reporting adhered to the standards set by the Preferred Reporting Items for Systematic Reviews and Meta-Analyses (PRISMA) statement [14] and followed the guidelines outlined in the Meta-Analyses of Observational Studies in Epidemiology (MOOSE) [15].

The patient population for this review consists of adult patients (18 years and above) who sustained a humeral shaft fracture secondary to trauma and subsequently underwent surgical fixation. The intervention is a surgical procedure involving the utilisation of intramedullary nailing devices, with inclusion given to both antegrade and retrograde entry points. The comparator is ORIF with plate fixation, encompassing all surgical approaches and incorporating minimally invasive plate osteosynthesis (MIPO). 

Search Strategy

A systematic literature search was conducted independently by both registered reviewers on medical databases including PubMed, Google Scholar, Embase, and Cochrane Central Register of Controlled Trials (CENTRAL). The date range included was from inception to June 2024, the search protocol was continued for new publications during the eligibility review of the compiled studies. The search was conducted using keywords: ‘humeral shaft fracture’, ‘diaphyseal humerus fractures’, ‘open reduction and internal fixation’ ‘ORIF’, ‘plate’, ‘intramedullary’, and ‘nail’. The keywords were expanded using synonyms and variations along with boolean operators to expand and specify the search. Both ‘free-text term’ and ‘Medical Subject Headings (MeSH) term’ were utilised. The search strategy is presented in Table 2 in the Appendices.

Additionally, backward citation tracking was performed manually by reviewing the reference lists of included studies to identify any further relevant articles that may have been missed during the initial search. Forward citation tracking was not conducted, as the aim of the meta-analysis was to focus on original research and primary articles retrieved directly from our predefined databases, ensuring relevance and methodological consistency. Finally, a grey literature search was conducted to review any relevant clinical guidelines or conference proceedings (opengrey.eu).

Screening Process and Eligibility Criteria

The compiled search results underwent a de-duplication process, where the EndNote reference management tool (Clarivate Analytics, Inc.) was used to automatically highlight potential duplicate records. These duplicates were then manually reviewed, confirmed, and removed to ensure accuracy. Studies were included if they reported on the primary or secondary outcomes of the review, comparing the surgical option in adult patients (18 years and above). Eligible study designs included randomized controlled trials (RCTs), cohort studies, case-control studies, and case series. Excluded were case reports, opinion articles, and studies not addressing the primary or secondary outcomes, those involving pathological fractures, paediatric populations, and fractures of the proximal and distal humerus. Only English-language articles were considered.

The principal reviewers independently applied the eligibility criteria to the search outcomes, resolving any discrepancies through discussion.

Data Extraction and Management

Data extraction and management were facilitated by the web-based tool Rayyan (Qatar Computing Research Institute, Ar-Rayyan, Qatar) [16]. Data extraction encompassed study location, dates, population demographics, surgical intervention details, as well as primary and secondary outcome measures as defined previously. Two reviewers compiled the information using a custom-designed datasheet in Microsoft Excel (Microsoft Corp., Redmond, USA). 

Data Analysis

The analysis of data adheres to data patterns associated with the investigated outcomes: (i) binary data, encompassing post-operative complications and iatrogenic injuries, were evaluated utilising the risk ratio; and (ii) continuous data, the time to fracture union, was assessed through the mean difference.

The execution of the meta-analysis and the generation of forest plots were conducted using Review Manager (RevMan) Web version 8.0.0 (Cochrane Collaboration, Oxford, UK) [17], incorporating the calculation of 95% confidence intervals. A random-effects model was employed given the heterogeneity of trauma patients. In the meta-analysis of observational studies, the MOOSE guidelines were adhered to [15].

Heterogeneity among the studies was scrutinised visually in a forest plot and statistically quantified using the I² test [18]. Significance was set at 40%.

Quality Appraisal and Bias Assessment

The quality assessment of the included studies was conducted using a standardised tool provided by the Joanna Briggs Institute (JBI). The updated checklist designed for RCTs, cohort studies, and case-control studies was employed for the critical appraisal of the included studies [19,20].

The evaluation of the risk of bias was independently performed by the two principal reviewers utilising the criteria outlined in the Cochrane Handbook for Systematic Reviews of Interventions [13], employing the following tools: (i) for RCTs, Risk of Bias in Randomized Trials (RoB-2) tool [21]; and (ii) for observational studies (cohort and case series), Risk of Bias in Non-Randomized Studies - of Interventions (ROBINS-I) tool [22]. Publication bias was assessed in the generated funnel plots on RevMan Web for symmetry under a random effects model. 

Any disparities in the assessment were resolved through discussion and consensus. The aggregated risk of bias assessments for the included studies is visually presented in a table for convenient reference.

Results

**Figure 1:**
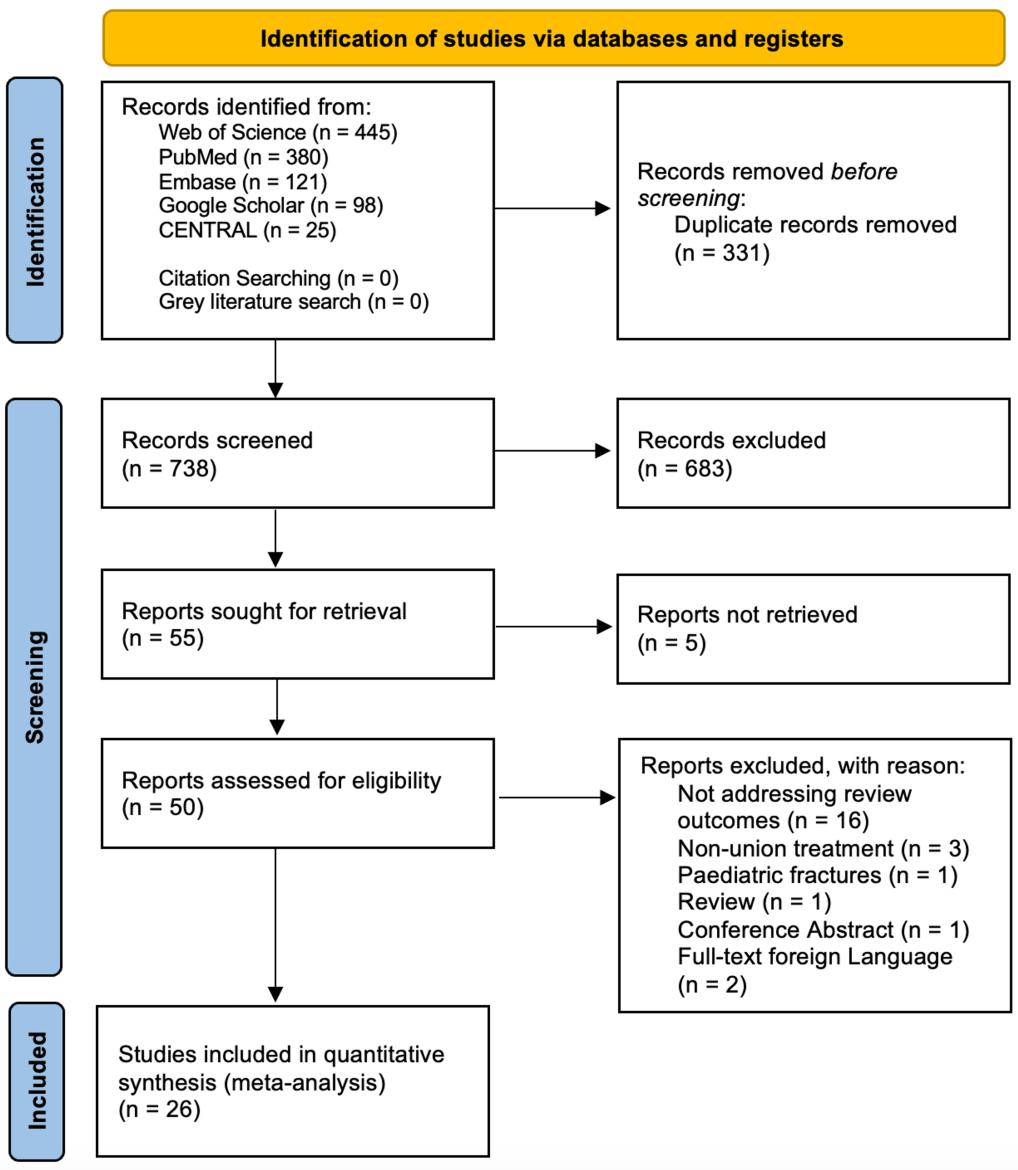
Preferred Reporting Items for Systematic Reviews and Meta-Analyses (PRISMA) diagram.

The search strategy initially identified 1069 articles. After de-duplication, 738 studies remained and were screened against the eligibility criteria. A total of 26 articles met the inclusion criteria. The process of study selection and attrition is detailed in the PRISMA flow chart presented in Figure 1.

Characteristics of Included Studies

Among the 26 included studies, there were 11 RCTs, one case-control study, six prospective cohort studies, and eight retrospective cohort studies. Collectively, these studies encompassed a total of 1674 patients, with 867 undergoing IMN and 807 ORIF. The cohort consisted of 65.2% males (1092) with a mean age of 41.7 years (IMN 43.5 years, ORIF 39.8 years). Follow-up periods varied widely ranging from four months to 130 months. 

**Figure 2:**
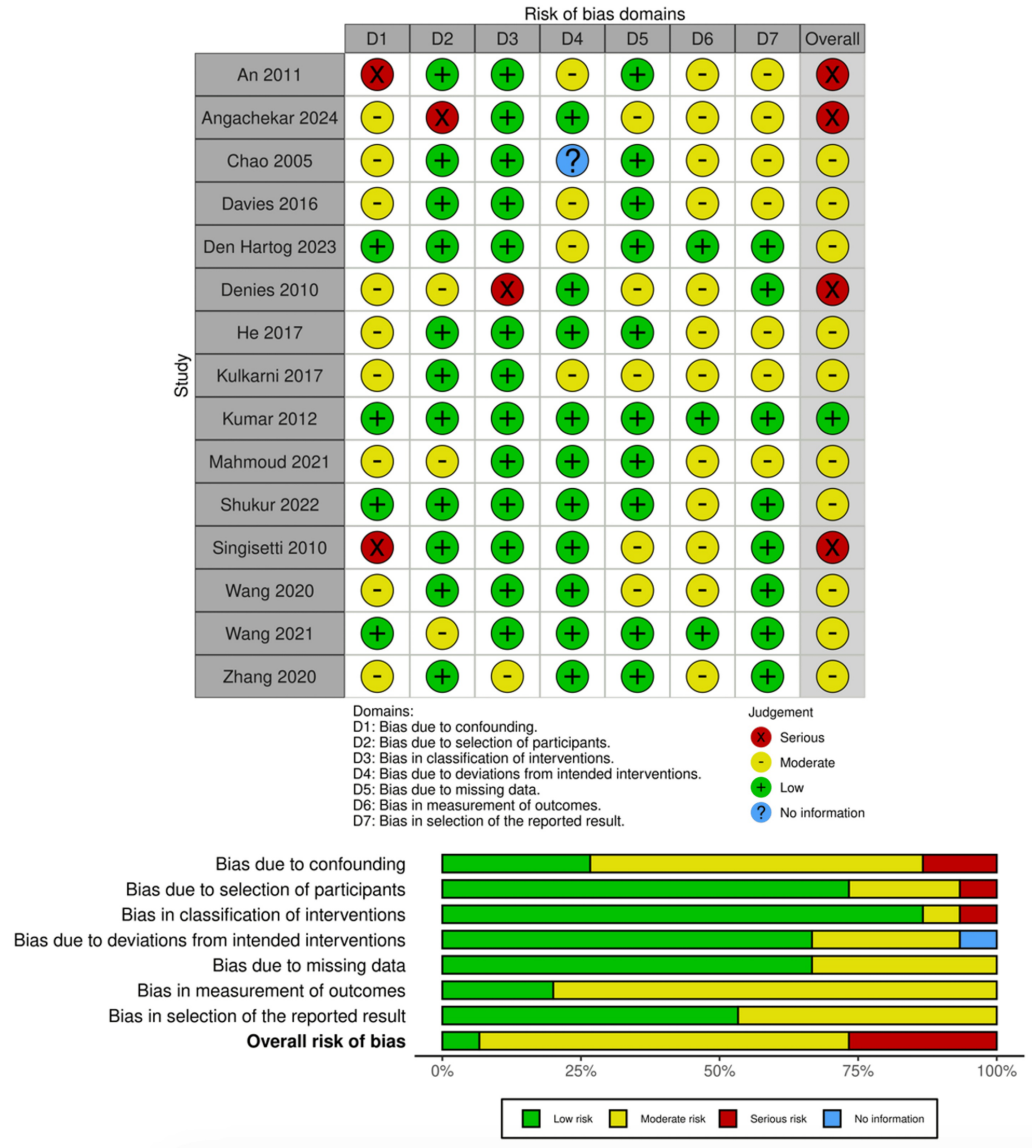
Risk of bias assessment of cohort and case-control studies using ROBINS-I tool. ROBINS-I tool [22,49] ROBINS-I: Risk of Bias in Non-Randomized Studies - of Interventions

**Figure 3:**
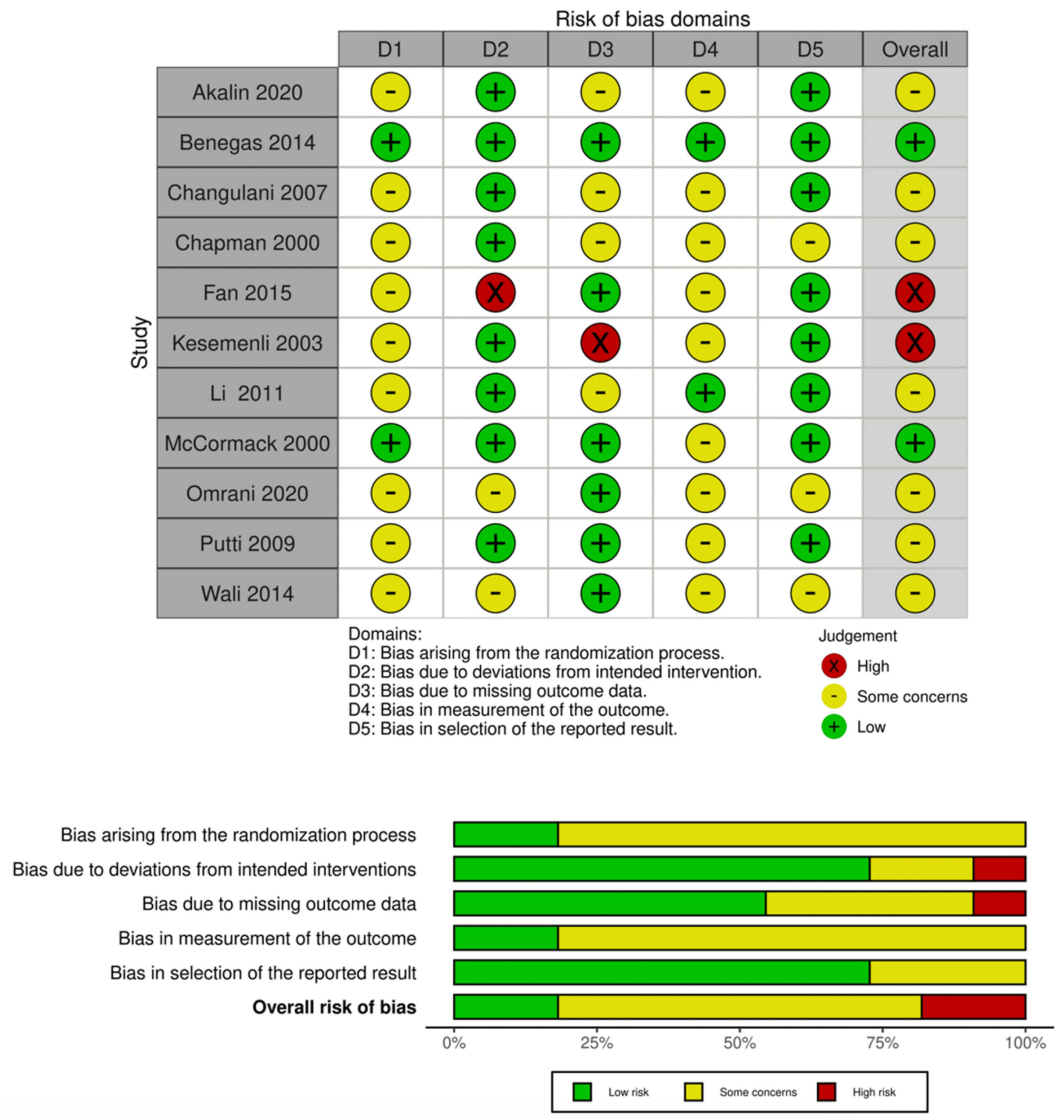
Risk of bias assessment of RCTs using RoB-2 tool. RoB-2 tool [21,49] RCT: Randomized controlled trial; RoB-2: Risk of Bias in Randomized Trials

The final compilation of included studies is summarised and presented in Table 1. The risk of bias assessment is presented in Figure 2 for studies screened with the ROBINS-I tool and Figure 3 for studies screened with the RoB-2 tool.

**Table 1:** Characteristics of included studies. * No breakdown of characteristics given. NR: Not reported; IMN: Intramedullary nailing; ORIF: Open reduction with internal fixation; M/F: Male/Female; RCT: Randomized controlled trial

Study ID and Author	Year	Methods	Location	N=		Mean Age (Age range), Years		Gender (M/F)		Mean Follow-up Period (Range), Months
				IMN	ORIF	IMN	ORIF	IMN	ORIF	
Akalin et al. [23]	2020	RCT	Turkey	30	33	41.5 (22-88)	45 (18-88)	17/13	24/9	24 (NR)
An et al. [24]	2011	Retrospective cohort	China	19	15	39.6 (19-62)	34.4 (21-51)	12/7	11/4	22.3 (12-62)
Angachekar et al. [25]	2024	Retrospective cohort	India	10	17	41.2 (18-60)	39.1 (25-72)	3/7	13/4	Minimum 4
Benegas et al. [26]	2014	RCT	Brazil	19	21	38.4 (NR)	44.8 (NR)	14/5	12/9	12 (NR)
Changulani et al. [27]	2007	RCT	India	21	24	39 (NR)	35 (NR)	20/3	19/5	14.3 (6-33)
Chao et al. [28]	2005	Retrospective cohort	Taiwan	56	36	49.3 (19-88)	53 (19-85)	33/23	20/16	71.9 (12-130)
Chapman et al. [29]	2000	RCT	USA	38	46	33 (18- 70)	34 (18-83)	26/12	25/21	14 (4-48)
Davies et al. [30]	2016	Retrospective Case-Control	Australia	15	15	46.7 (20-85)	48.0 (16-94)	10/5	9/6	12
Den Hartog et al. [31]	2023	Prospective cohort	Netherlands	169	76	Median 57 (NR)	Median 43 (NR)	74/95	38/38	12 (12-14)
Denies et al. [32]	2010	Retrospective cohort	Belgium	49	42	52.4 (NR)	48.2 (NR)	21/28	25/17	24 (NR)
Fan et al. [33]	2015	RCT	China	30	30	39.3 (NR)	39.2 (NR)	18/12	19/11	12 (NR)
He et al. [34]	2017	Retrospective cohort	China	54	58	30.9 (20-41)	30.5 (18-44)	40/14	38/20	14.7 (6-24)
Kesemenli et al. [35]	2003	RCT	Turkey	33	27	42 (21-61)	33 (19-47)	24/9	19/8	42 (28-72)
Kulkarni et al. [36]	2017	Retrospective cohort	India	44	68	39.6 (18-70) Both groups*		83/29 Both groups*		6 (NR)
Kumar et al. [37]	2012	Prospective cohort	India	15	15	46.1 (NR)	44.7 (NR)	8/7	10/5	16 (16-19)
Li et al. [38]	2011	RCT	China	22	23	39.9 (20-60)	35.7 (20-60)	16/6	16/7	12 (NR)
Mahmoud et al. [39]	2021	Prospective cohort	Egypt	21	21	34.8 (NR)	38.5 (NR)	16/5	15/6	29.9 (NR)
McCormack et al. [40]	2000	RCT	Canada	21	23	40 (19-82)	49 (20-81)	13/8	15/8	14.3 (6-33)
Omrani et al. [41]	2020	RCT	Iran	40	40	31.3 (NR)	29.8 (NR)	25/15	22/18	12 (NR)
Putti et al. [42]	2009	RCT	India	16	18	36 (23-84)	39 (22-65)	32/2	32/2	24 (NR)
Shukur et al. [43]	2022	Prospective cohort	Iraq	15	15	41.1 (19-64) Both groups*		10/5	10/5	6 (NR)
Singisetti et al. [44]	2010	Prospective cohort	UK	20	16	(18-63) Both groups*		28/8 Both groups*		12 (10-24)
Wali et al. [45]	2014	RCT	India	25	25	37.3 (NR)	37.7 (NR)	21/4	20/5	12 (NR)
Wang et al. [46]	2020	Prospective cohort	China	26	27	45.7 (20-79)	45.4 (18-88)	16/10	17/13	12 (NR)
Wang et al. [47]	2021	Retrospective cohort	China	25	30	39.3 (18-56)	37.2 (18-56)	16/9	17/13	12 (NR)
Zhang et al. [48]	2020	Retrospective cohort	China	34	46	43.2 (NR)	41.0 (NR)	19/15	31/15	24 (NR)

*Time to Union* 

**Figure 4:**
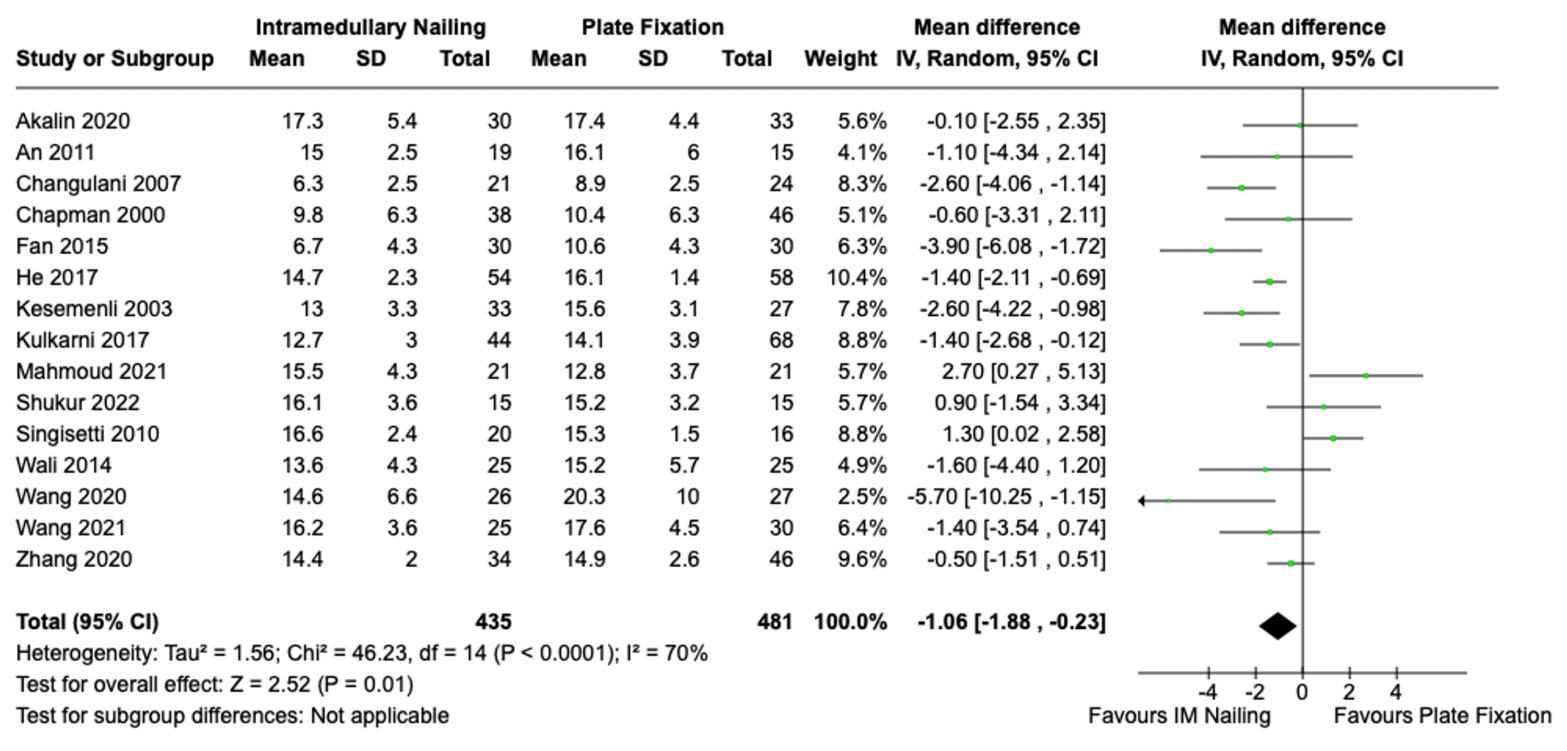
Time to union IMN vs. ORIF with plate fixation. References: Akalin et al. [23], An et al. [24], Changulani et al. [27], Chapman et al. [29], Fan et al. [33], He et al. [34], Kesemenli et al. [35], Kulkarni et al. [36], Mahmoud et al. [39], Shukur et al. [43], Singisetti et al. [44], Wali et al. [45], Wang et al. 2020 [46], Wang et al. 2021 [47], Zhang et al. [48] IMN: Intramedullary nailing; ORIF: Open reduction with internal fixation

The time to union was reported in a total of 15 studies, encompassing 435 patients who underwent IMN and 481 patients who received ORIF. The average time to union for IMN was 13.3 weeks and for ORIF it was 14.6 weeks. This was a statistically significant difference in favour of IMN with a mean difference of -1.06 (95% CI, -1.88 to -0.23, Z = 2.52, p = 0.01). There was significant statistical heterogeneity among the studies I^2^ = 70% (p < 0.0001). The forest plot is presented in Figure 4. 

Time to Union (RCTs)

**Figure 5:**
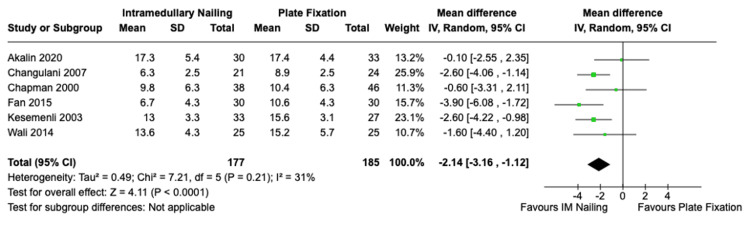
Time to union IMN vs. ORIF with plate fixation - sub-group analysis of RCTs. References: Akalin et al. [23], Changulani et al. [27], Chapman et al. [29], Fan et al. [33], Kesemenli et al. [35], Wali et al. [45] IMN: Intramedullary nailing; ORIF: Open reduction with internal fixation; RCT: Randomized controlled trial

A sub-group analysis was completed of the included RCT studies reporting time to union, totalling six trials, encompassing 177 patients who underwent IMN and 185 patients who received ORIF. The average time to union for IMN was 11.3 weeks and for ORIF it was 12.9 weeks. This was a statistically significant difference in favour of IMN with a mean difference of -2.14 (95% CI, -3.16 to -1.12, Z = 4.11, p < 0.00011). There was no significant statistical heterogeneity among the studies I^2^ = 31% (p = 0.21). The forest plot is presented in Figure 5.

Non-union

**Figure 6:**
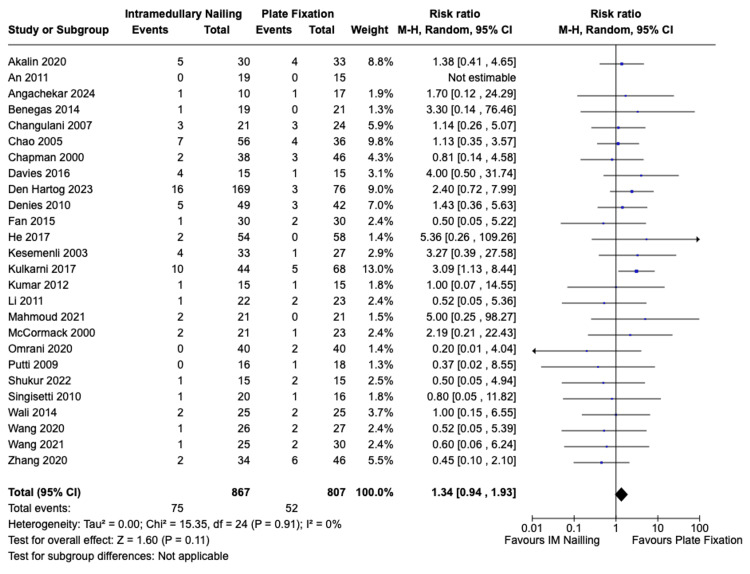
Risk of non-union IMN vs. ORIF with plate fixation. References: Akalin et al. [23], An et al. [24], Angachekar et al. [25], Benegas et al. [26], Changulani et al. [27], Chao et al. [28], Chapman et al. [29], Davies et al. [30], Den Hartog et al. [31], Denies et al. [32], Fan et al. [33], He et al. [34], Kesemenli et al. [35], Kulkarni et al. [36], Kumar et al. [37], Li et al. [38], Mahmoud et al. [39], McCormack et al. [40], Omrani et al. [41], Putti et al. [42], Shukur et al. [43], Singisetti et al. [44], Wali et al. [45], Wang et al. 2020 [46], Wang et al. 2021 [47], Zhang et al. [48] IMN: Intramedullary nailing; ORIF: Open reduction with internal fixation

The non-union incidence was reported in all 26 included studies, encompassing 867 patients who underwent IMN and 807 patients who received ORIF. The incidence of non-union for IMN was 75 (8.7%) and for ORIF it was 52 (6.4%). There was no statistically significant difference in favour of either fixation modality with a risk ratio of 1.34 (95% CI, 0.94 to 1.93, Z = 1.6, p = 0.11). There was no significant statistical heterogeneity among the studies I^2^ = 0% (p = 0.91). The forest plot is presented in Figure 6. 

Iatrogenic Radial Nerve Palsy

**Figure 7:**
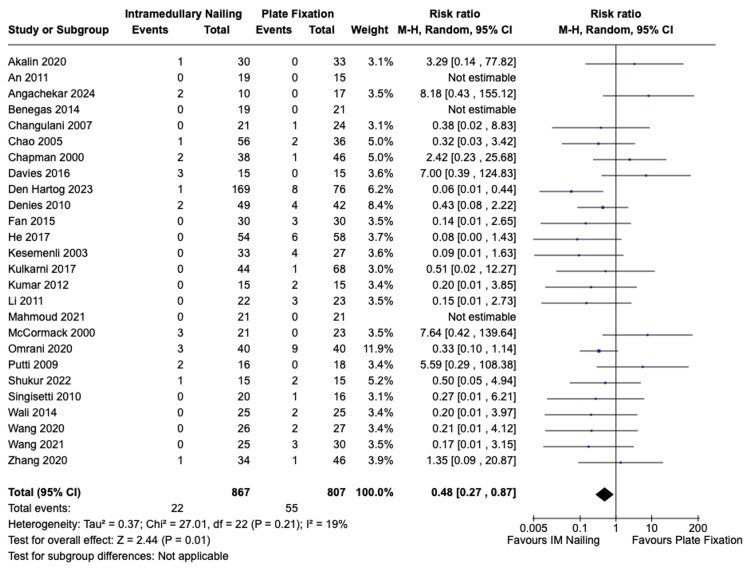
Risk of iatrogenic radial nerve injury IMN vs. ORIF with plate fixation. References: Akalin et al. [23], An et al. [24], Angachekar et al. [25], Benegas et al. [26], Changulani et al. [27], Chao et al. [28], Chapman et al. [29], Davies et al. [30], Den Hartog et al. [31], Denies et al. [32], Fan et al. [33], He et al. [34], Kesemenli et al. [35], Kulkarni et al. [36], Kumar et al. [37], Li et al. [38], Mahmoud et al. [39], McCormack et al. [40], Omrani et al. [41], Putti et al. [42], Shukur et al. [43], Singisetti et al. [44], Wali et al. [45], Wang et al. 2020 [46], Wang et al. 2021 [47], Zhang et al. [48] IMN: Intramedullary nailing; ORIF: Open reduction with internal fixation

Iatrogenic radial nerve palsy incidence was reported in all 26 included studies, encompassing 867 patients who underwent IMN and 807 patients who received ORIF. The incidence of post-operative radial nerve palsy for IMN was 22 (2.5%) and for ORIF it was 55 (6.8%). There was a statistically significant difference in favour of IMN with a risk ratio of 0.48 (95% CI, 0.27 to 0.87, Z = 2.44, p = 0.01). There was no significant statistical heterogeneity among the studies I^2^ = 19% (p = 0.21). The forest plot is presented in Figure 7. 

Surgical Site Infection

**Figure 8:**
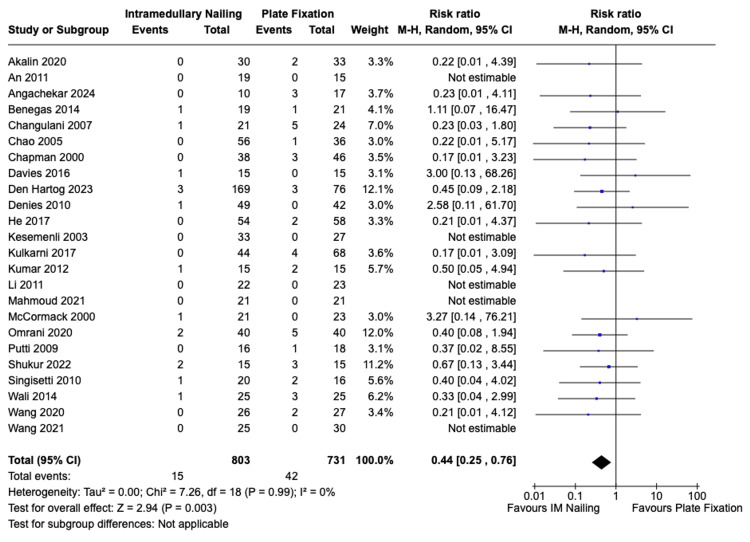
Risk of surgical site infection IMN vs. ORIF with plate fixation. References: Akalin et al. [23], An et al. [24], Angachekar et al. [25], Benegas et al. [26], Changulani et al. [27], Chao et al. [28], Chapman et al. [29], Davies et al. [30], Den Hartog et al. [31], Denies et al. [32], He et al. [34], Kesemenli et al. [35], Kulkarni et al. [36], Kumar et al. [37], Li et al. [38], Mahmoud et al. [39], McCormack et al. [40], Omrani et al. [41], Putti et al. [42], Shukur et al. [43], Singisetti et al. [44], Wali et al. [45], Wang et al. 2020 [46], Wang et al. 2021 [47] IMN: Intramedullary nailing; ORIF: Open reduction with internal fixation

Surgical site infection was reported in a total of 24 studies, encompassing 803 patients who underwent IMN and 731 patients who received ORIF. The incidence of post-operative surgical site infection for IMN was 15 (1.9%) and for ORIF it was 42 (5.7%). There was a statistically significant difference in favour of IMN with a risk ratio of 0.44 (95% CI, 0.25 to 0.76, Z = 2.94, p = 0.003). There was no significant statistical heterogeneity among the studies I^2^ = 0% (p = 0.99). The forest plot is presented in Figure 8.

Intra-operative Comminution

**Figure 9:**
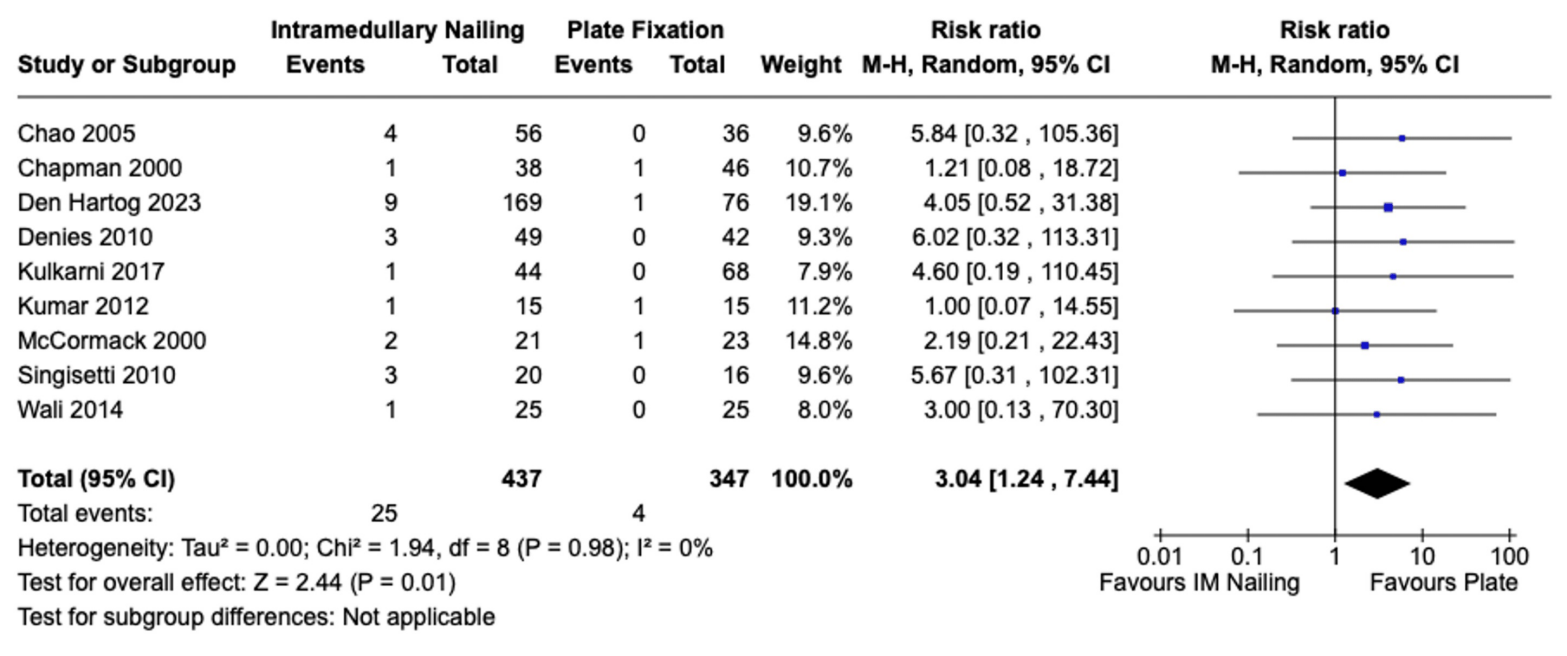
Risk of intra-operative comminution IMN vs. ORIF with plate fixation. References: Chao et al. [28], Chapman et al. [29], Den Hartog et al. [31], Denies et al. [32], Kulkarni et al. [36], Kumar et al. [37], McCormack et al. [40], Singisetti et al. [44], Wali et al. [45] IMN: Intramedullary nailing; ORIF: Open reduction with internal fixation

Intra-operative comminution at the fracture site was reported in nine of the included studies, encompassing 437 patients who underwent IMN and 347 patients who received ORIF. The incidence of intra-operative comminution for IMN was 25 (5.7%) and for ORIF it was 4 (1.2%). There was a statistically significant difference in favour of ORIF with a risk ratio of 3.04 (95% CI, 1.24 to 7.44, Z = 2.44, p = 0.01). There was no significant statistical heterogeneity among the studies I^2^ = 0% (p = 0.98). The forest plot is presented in Figure 9.

Discussion

This systematic review aimed to compare the outcomes of IMN versus ORIF in the management of humeral shaft fractures. The comprehensive analysis of various studies provides an understanding of the effectiveness, risks, and benefits associated with these two surgical techniques.

Review Outcomes

The primary outcome of this review was time to union, with results indicating a shorter time to union for IMN compared to ORIF. Despite significant statistical heterogeneity in the reported results, likely due to variations in the definitions of union time and the inherent heterogeneity of the trauma patient population. Sub-group analysis of RCTs showed lower statistical heterogeneity and continued to demonstrate faster time to union with IMN, reinforcing the notion that IMN may provide a more rapid bony union. However, the absolute difference in union times, while statistically significant, may not translate into a substantial improvement in clinical outcomes.

The review found no significant difference in non-union rates between IMN and ORIF, indicating that both techniques are effective in achieving bone union. This demonstrates the importance of considering other factors, such as patient comorbidities and surgeon expertise when choosing the fixation modality. The studies reviewed often lacked appropriate blinding and surgeon preference might have introduced bias, as patients at higher risk of non-union were more likely to receive ORIF to ensure anatomical reduction with absolute stability to mitigate this risk [7,50].

Iatrogenic radial nerve palsy and surgical site infections were more prevalent in the ORIF group. The extensive soft tissue dissection required overlying the radial nerve and longer operation times likely contribute to these higher rates [1,50]. Most radial nerve injuries in the ORIF group were neuropraxic and resolved without intervention, whereas IMN-related nerve injuries, though less frequent, were often more severe due to entrapment within the fracture site or injury during distal locking screw insertion [51,52].​ The lower rate of surgical site infections associated with IMN's minimally invasive approach would make it particularly beneficial for patients with higher infection risks, such as those with diabetes or immunosuppression. 

IMN was associated with a higher risk of intra-operative comminution, likely due to the stress concentration from canal reaming and the risks of closed reduction attempts. The risk of bias in these findings remains high, given the potential patient selection bias, as IMN would be preferred in patients with poor bone quality due to its load-sharing construct.

Clinical Implications

Functional outcomes, surgical risks, and operation time are critical considerations in the choice of fixation. IMN generally requires shorter operation times compared to ORIF, reducing anaesthesia-related risks [53,54]. Additionally, it offers faster union times and a minimally invasive approach, which lowers rates of blood loss, radial nerve palsy, and surgical site infections [54,55]. The load-sharing construct and preservation of fracture biology further enhance IMN's suitability in patients requiring early rehabilitation and minimisation of surgical risks. This highlights IMN as advantageous for poly-traumatised patients by aligning with the principles of Damage Control Orthopaedics (DCO) by minimising the “second hit” on the patient’s physiology [56]. Moreover, it is beneficial for elderly frail patients with poor bone stock. Although shoulder complications remain a concern with antegrade IMN insertions, these issues have been somewhat mitigated with new-generation nail designs [57,58].

Comparatively, ORIF offers the advantage of achieving anatomical reduction and lower re-operation rates by allowing for precise control over the length, alignment, and rotation of the limb, which is crucial in reducing cosmetic deformity and preventing malunion [1]. Although malunion may be functionally tolerated in the upper limb [3], its presence can significantly impact both aesthetic and functional outcomes, especially in younger and more active patients requiring optimal limb function. These patients are less likely to tolerate upper-limb deformities, which can adversely affect their quality of life, psychological well-being, and financial income [59,60].

The wide variety of patient-reported outcome measures (PROMs), as well as the varying timelines for testing and significant potential for assessor bias present in the current literature, have made it difficult to conduct any meaningful analysis of PROMs across different studies. Leading to primarily objective measure analyses leaving a key area of post-operative outcomes unaccounted for.

Both IMN and ORIF have advantages and limitations, and their optimal use depends on a comprehensive assessment of patient needs, fracture characteristics, and potential risks. Continued advancements in fixation designs and ongoing research will further refine these treatment modalities, enhancing patient outcomes in the management of humeral shaft fractures.

Future Research

Despite the insights provided by this review, significant risks of bias, varying clinical practices, and variation in surgical technique over time limit the ability to draw firm conclusions. This highlights the need for large multicentre randomized trials to provide more robust evidence. The ongoing Humeral Shaft Fracture (HUSH) Trial comparing non-operative ORIF and IMN is expected to contribute valuable data to this field, offering further clarity and context to the current knowledge base​​​ [61]. Future research should focus on standardising outcome measures, long-term functional outcomes, and quality-of-life assessments to better guide clinical decision-making and improve patient care.

Limitations

This review has several limitations that may impact the generalisability of its findings. The heterogeneity among the study designs, including variable follow-up durations, outcome measures, and definitions such as time to union, complicates the ability to draw consistent conclusions. The majority of included studies lacked randomization, and the RCTs that were included often had issues related to the randomization process, introducing a significant risk of selection bias.

There is also considerable clinical heterogeneity across the included studies due to the wide variation in trauma patients, with limited evidence of robust control for confounding factors. Additionally, the retrospective nature of many trauma studies further compounds the issue.

Quality appraisal was conducted using the JBI standardised checklist tool, revealing methodological flaws in all included studies. However, these flaws were not severe enough to warrant exclusion from the review. The risk of bias assessment, performed using the RoB-2 and ROBINS-I tools, indicated an overall moderate risk of bias in the available literature. This was primarily due to issues with randomization and outcome measurement in RCTs and confounding factors and outcome measurement in cohort studies.

Despite these limitations, the pooled analysis of objective outcomes from 1674 patients represents a substantial contribution relative to the typically small sample sizes and single-centre designs prevalent in this research area. While the review provides valuable insights, the findings should be interpreted with caution due to these inherent limitations.

## Conclusions

IMN demonstrated a shorter time to union, potentially facilitating faster recovery, though the absolute clinical significance is modest. Both techniques effectively achieved bone union, with no significant difference in non-union rates. IMN was associated with lower incidences of iatrogenic radial nerve palsy and surgical site infections. Significant heterogeneity, variable outcome measures, and potential biases highlight the need for caution in interpreting results. Given these limiting factors, this review recommends favouring IMN in situations where rapid stabilisation is required with a minimal additional insult to the patient, particularly in poly-traumatised and frail patient groups. Outside of these situations, ORIF should be the preferred fixation modality due to its advantages in achieving precise anatomical reduction and preventing union issues.

## Appendices

**Table 2:** Electronic database search strategy. MeSH: Medical Subject Headings

	AND		
	Patient (Component 1)	Intervention (Component 2)	Comparator (Component 3)
OR	Humerus shaft fracture	Intramedullary nail	Open reduction internal fixation
	Humerus diaphyseal fracture	IM nail	Plating
	Diaphyseal humerus fracture	Locking nail	Locking plate
	Humer* shaft fracture	Intramedullary device	Compression plate
	Humer* diaphyseal fracture		Plat*
MeSH Terms	humerus fractures, bone diaphyses humeral fractures	fracture fixation, intramedullary nails	fracture fixation, internal bone plates
